# Supplementary material for: Resistance exercise interventions for sarcopenia and nutritional status of maintenance hemodialysis patients: a meta-analysis
Source: PeerJ. 2024 Feb 5;12:e16909. doi: 10.7717/peerj.16909 (PMC10851873; doi:10.7717/peerj.16909)
Supplement: Supplemental Information 3 [file peerj-12-16909-s003.docx]

**Retrieval strategy**

**PubMed**:

((((((maintenance hemodialysis[Title/Abstract]) OR (maintenance dialysis[Title/Abstract])) OR (hemodialysis[Title/Abstract])) OR (dialysis[Title/Abstract])) OR (blood dialysis[Title/Abstract])) OR (MHD[Title/Abstract])) AND (((((((resistance training[Title/Abstract]) OR (resistance exercise[Title/Abstract])) OR (resistive exercise[Title/Abstract])) OR (strength training[Title/Abstract])) OR (strength-type training[Title/Abstract])) OR (strength-type exercise[Title/Abstract])) OR (physical training[Title/Abstract]))

**EMBASE:**

#1 'maintenance hemodialysis OR 'maintenance dialysis OR hemodialysis OR 'dialysis' OR 'blood dialysis OR 'mhd'

#2 'resistance training' OR 'resistance exercise' OR resistive exercise' OR 'strength training' OR 'strength-type training' OR 'strength-type exercise 'physical training'

#3 #1 AND #2

**Cochrane Library:**

#1 (maintenance hemodialysis): ti, ab,kw

#2 (maintenance dialysis):ti,ab,kw

#3 (hemodialysis):ti.ab,kw

#4 (dialysis):ti,ab, kw

#5 (blood dialysis): tab,kw

#6 (MHD): tab,kw

#7 #1 OR #2 OR #3 OR #4 OR #5 OR #6

#8 (resistance training):ti,ab,kw

#9 (resistance exercise):ti,ab.kw

#10 (resistive exercise): ti,ab.kw

#11 (strength training): ti, ab,kw

#12 (strength-type training): ti, ab,kw

#13 (strength-type exercise):tl, ab,kw

#14 (physical training): ti,ab,kw

#15 #8 OR #9 OR #10 OR #11 OR #12 OR #13 OR #14

#16 #7 AND #15

**Web of Science:**

#1 (((((TS=(maintenance hemodialysis)) OR TS=(maintenance dialysis)) OR TS=(hemodialysis)) OR TS=(dialysis)) OR TS=(blood dialysis)) OR TS=(MHD)

#2

((((((TS=(resistance training)) OR TS=(resistance exercise)) OR TS=(resistive exercise)) OR TS=(strength training)) OR TS=(strength-type training)) OR TS=(strength-type exercise)) OR TS=(physical training)

#3 #1 AND #2

**CNKI:**

SU: maintenance hemodialysis OR hemodialysis

SU: resistance training OR resistance exercise Search in results

Wan Fang: (maintenance hemodialysis OR hemodialysis) AND (resistance training OR resistance exercise )

VIP: U:( maintenance hemodialysis OR hemodialysis) AND (resistance exercise OR resistance training)

**CBM:**

#1 maintenance hemodialysis

#2 hemodialysis

#3 #1 OR #2

#4 resistance exercise

#5 resistance training

#6 #4 OR #5

#7 #3 AND #6
